# Strength and Toughness of Waste Fishing Net Fiber-Reinforced Concrete

**DOI:** 10.3390/ma14237381

**Published:** 2021-12-02

**Authors:** Tri N. M. Nguyen, Taek Hee Han, Jun Kil Park, Jung J. Kim

**Affiliations:** 1 Campus in Ho Chi Minh City, University of Transport and Communications, No. 450-451 Le Van Viet Street, Tang Nhon Phu A Ward, Thu Duc City, Ho Chi Minh City 700000, Vietnam; trinnm_ph@utc.edu.vn; 2 Coastal Development and Ocean Energy Research Center, Korea Institute of Ocean Science and Technology, 385 Haeyang-ro, Yeongdo-gu, Busan 49111, Korea; taekheehan@kiost.ac.kr (T.H.H.); jkpark@kiost.ac.kr (J.K.P.); 3 Department of Civil Engineering, Kyungnam University, 7 Kyungnamdaehakro, Changwon-si 51767, Korea

**Keywords:** waste fishing net fiber, recycled material, concrete, strength, toughness, biaxial flexure

## Abstract

In this study, we estimate the potential efficiency of waste fishing net (WFN) fibers as concrete reinforcements. Three WFN fiber concentrations (1, 2, and 3% by volume) were mixed with concrete. Compressive strength, toughness, splitting tensile strength, and biaxial flexural tests were conducted. Compressive strength decreased but other properties increased as a function of fiber proportions. According to the mechanical strength observations and the ductility number, WFN fibers yielded benefits in crack arresting that improved the postcracking behavior and transformed concrete from a brittle into a quasi-brittle material. It is inferred that WFN fiber is a recycled and eco-friendly material that can be utilized as potential concrete reinforcement.

## 1. Introduction

The civil construction industry has contributed significantly to development worldwide. However, drawbacks still exist. In particular, the consumption of mineral resources and fossil fuels in conjunction with the huge amounts of waste generated has affected the environment. According to [1], in 2019, CO_2_ emissions and energy consumptions caused by the construction sector were recorded at 38 and 35%, respectively, as compared with the proportions pertaining to the entire industry. In addition, the environment is worsening owing to an increase in marine litter. According to [2], the growth of annual marine plastic litter is predicted to increase from 11 million metric tons in 2016 to 29 million metric tons in 2040. Almost all marine litter is made from poor or non-degradable materials; thus, it is harmful to the ecosystem. One of the highest volume components that can be easily found along shorelines or floating on the ocean surface is waste fishing net (WFN). WFNs are generally made from nonbiodegradable materials that are advantageous in fisheries. However, they are harmful to the ecosystem when out of service. In particular, the use of WFNs constitutes one of the causes of marine accidents because of their entanglement around ships’ propellers. Thus, the use of fibers from recycled WFNs to reinforce concrete is a potential solution [3,4,5,6]. If successful, it can solve both the environmental and economic issues.

Synthetic polymeric fibers used in cement-based materials can be observed in micro- or macroforms. Our previous studies showed the effectiveness of electrospun nanofibers in increasing the tensile strength and toughness of cement materials [7,8,9,10]. From the literature reviews, numerous types of macrofibers have been utilized to strengthen concrete with high performance. The changes in cement-based material characteristics, such as compressive, splitting tensile, direct tensile and flexural strengths, modulus of rupture, impact, shrinkage, and freeze–thaw resistance, have been reported following the addition of different types of synthetic fibers in concrete, such as polypropylene (PP) [11,12,13,14,15,16,17], polyethylene terephthalate (PET) [18,19], polyvinyl alcohol (PVA) [17,20], high-density polyethylene (HDPE) [21], polybenzoxazoles (PBO) [21], nylon [11], and polyethylene (PE) [22]. The potential results of reinforcing cement-based materials using synthetic polymeric fibers have been observed worldwide. Many efforts have been made to strengthen concrete using recycled fibers from these polymeric materials. Zhang et al. [17] clarified the effectiveness of recycled PP fibers (R-PP) on the workability and mechanical properties of geopolymer concrete. Their observations showed a decrease in compressive strength and an increase in splitting tensile and flexural strengths when R-PP fibers (1% by volume) were added to concrete. However, all of the above mechanical properties increased with the addition of the same amount of hybrid fibers, which contained steel and R-PP fibers. The observation from this study also showed a change in the failure mode from brittle to ductile regarding the composite materials. In [23], the performance of recycled HDPE fibers (R-HDPE) was changed as manifested by improvements in tensile strength, flexural toughness, and durability, and minor reductions in compressive strength and modulus of elasticity of concrete. Kim et al. [24] proposed a process for fabricating recycled PET fibers (R-PET) from bottle waste. In addition, attempts were made to clarify the influences of R-PET fibers on the improvement in free drying shrinkage strain, tensile strength, and ductility and the degradation of the compressive behaviors of concrete. The effectiveness of R-PET fibers in reducing the cost of the material while achieving good energy absorption capacity in cementitious composites can be found in [25]. In addition, the study of Tran et al. [26] dealt with the performance of pervious concrete containing fibers obtained from recycled plastic bags. Their observations showed an improvement in the microparticle filtration capacity while both the compressive and flexural strengths of the composite materials were reduced. Orasutthikul et al. [27] compared the mechanical behavior of cement-based materials modified with recycled nylon fibers (R-nylon) from WFNs with those modified by recycled PET (R-PET) and PVA fibers. Similar to the other studies concerned with reinforcing cement-based materials with synthetic polymeric fibers, a decrease in compressive behavior was observed when these recycled fibers were added into the mortar. However, observations from this study showed the effectiveness of R-nylon fibers from WFNs in improving the flexural strength, material toughness, and residual strength of mortar. Spadea et al. [28] also observed the effectiveness of R-nylon fibers obtained from WFNs as a tensile reinforcement for mortars. Notably, all of the above synthetic polymeric materials have been used to produce fishing nets owing to their high durability and high resistance to tensile and impact loading [29,30,31,32].

The observations of previous studies revealed the feasibility of using recycled PE fibers (R-PE) from WFNs as tensile reinforcement for mortar [14,32]. However, systematic experimental approaches used to estimate the mechanical properties of concrete that contains R-PE fibers from WFNs are lacking. Therefore, further information on the mechanical characteristics of WFN fiber-reinforced concrete is required. To address these gaps in the literature, this study has been conducted to: (1) present a feasibility approach for using the fibers produced from WFNs as reinforcement agents for normal concrete to reduce environmental issues; (2) investigate and estimate the mechanical behavior and failure mode of concrete that has been reinforced by different proportions of WFN fibers by using a compressive strength test (American Society for Testing and Materials (ASTM) C39) [33], splitting tensile strength test (ASTM C496) [34], and biaxial flexure test (ASTM C1550) [35]; (3) determine the appropriate fiber proportion for observing the best mechanical performance, postcracking behavior, and economic benefit of WFN fiber-reinforced concrete.

## 2. Experimental Work

### 2.1. Materials

**Figure 1:**
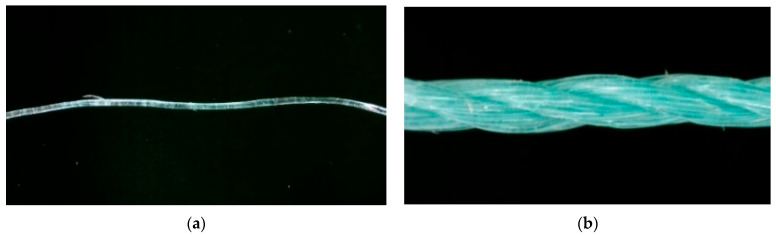
Geometry of the waste fishing net (WFN) fibers: (**a**) Filament; (**b**) fiber.

**Figure 2:**
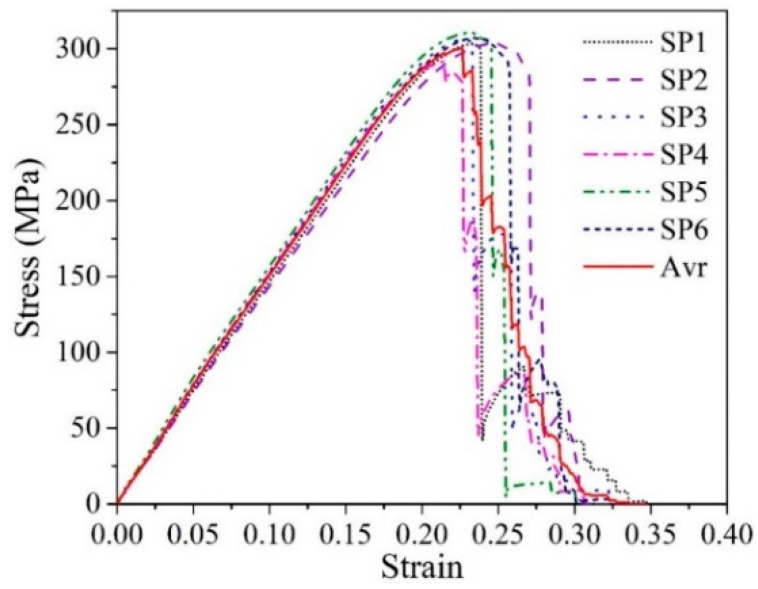
Tensile testing result of the WFN fibers.

In this study, WFN fibers (or R-PE fibers) were used as reinforcement agents for normal concrete. The twisted multifilament R-PE fibers were produced from WFNs collected alongside the seashore of Ulsan, Korea. The production process has already been reported [14,32]. Figure 1 and Figure 2 and Table 1 present the properties of the WFN fiber. Table 2 presents the difference in tensile strength of the present WFN fibers as compared with that of the commercial PE fibers and other recycled polymeric fibers in the literature. As compared with the tensile strength of commercial PE fiber presented in [36], the tensile strength of the WFN fibers was reduced by 24%. However, as compared with other studies on reinforced cement-based materials using recycled polymeric fibers [17,24,28], a comparative study of the tensile strength of WFN fibers can be conducted.

Table 3 presents the mixture designs for the plain concrete and the WFN fiber-reinforced concrete. In this present study, the chosen WFN fiber proportions were 1, 2, and 3% by volume of plain concrete. The chemical compositions and physical properties of the cement are listed in Table 4.

### 2.2. Sample Preparations

**Figure 3:**
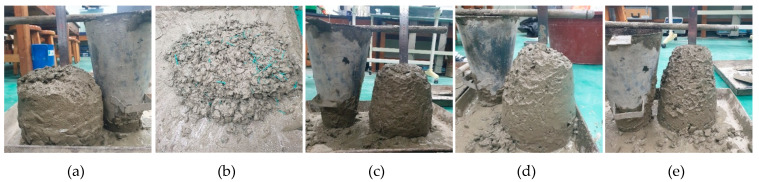
(**a**) Slump test for plain mix; (**b**) fresh wet mix of WFN fiber-reinforced concrete. Slump test for fresh mix of WFN fiber-reinforced concrete: (**c**) 1% WFN fiber-reinforced concrete; (**d**) 2% WFN fiber-reinforced concrete; (**e**) 3% WFN fiber-reinforced concrete.

A mixture design was proposed to observe the constituents of conventional concrete, which expected a compressive strength at 28 days of 40 MPa (refer to Table 3). The water-to-cement ratio (W/C) was consistently set to 0.45. The process of preparing the samples for estimating the hardened characteristics of WFN fiber-reinforced concrete was conducted as follows: The plain concrete mixes were prepared with a concrete mixer based on a normal process [18,26]. The slump tests were conducted to ensure consistency in the workability characteristics for all the plain batches before the specified amounts of WFN fibers were added [37]. The average slump was 11 cm. Notably, the slump tests were reconducted with the as-mixed batches that contained WFN fibers to estimate the workability of the WFN fiber-reinforced concrete materials. Then, the specified amounts of WFN fibers were added to each plain batch, and hand mixing was conducted using shovels. Finally, the as-mixed batches were poured into D100 × 200 mm^2^ cylindrical molds and D420 × 48 mm^2^ round panel molds, compacted with a steel rod [38]. Notably, owing to the twisted geometry of the fibers, the filament-separated phenomenon would occur if we executed the fiber mixing process using the mixer. In this study, there were 24 cylindrical samples prepared for the compressive strength and splitting tensile strength tests, and nine round panel samples were prepared for the biaxial flexure test (see Table 5). All specimens were cured in water for 28 days in standard laboratory conditions (20 ± 20 °C, 50 ± 5% relative humidity (RH)). Plain concrete was also prepared as a reference sample. Figure 3 presents the slump test and fresh wet mix of the WFN fiber-reinforced concrete batch.

## 3. Testing Methods

In this study, the slump test was conducted in compliance with ASTM C143 [37] for all fresh and modified concrete batches. Two equal layers of concrete were poured into the cone mold and compacted 25 times per layer. The slump test was conducted to ensure similar workability of the fresh concrete before the addition of WFN fibers and to estimate the workability of the WFN fiber-reinforced concrete materials. The compressive and splitting tensile strength tests were conducted using a hydraulic universal testing machine (hydraulic UTM) with a capacity of 1000 kN according to the specifications of ASTM C39 [33] and ASTM C496 [34], respectively. Two linear variable differential transformers (LVDTs) were attached to measure the strain and deformation for the compressive strength and splitting tensile strength tests, respectively. Four variable groups, including plain concrete, 1, 2, and 3% WFN fiber-reinforced concrete, each of which contained three cylindrical samples, were conducted for each test. There was a total of 24 cylindrical samples used in this study (see Table 5). The mean values of the three samples of the compressive strength and splitting tensile strength were calculated using Equations (1) and (2), respectively, as follows:

(1) $$f_{c}=\frac{P}{A}$$

(2) $$f_{sp}=\frac{2P}{\pi LD}$$

where *f_c_* and *f_sp_* are the compressive and splitting tensile strengths (MPa), respectively; *P* is the maximum applied load of each test (kN); *A* is the area of the cross-section of the cylinder (mm^2^); and *L* and *D* represent the cylindrical sample’s length and diameter (mm).

The equibiaxial flexural stress and the toughness of the WFN fiber-reinforced concretes were estimated according to the novel biaxial flexure test (BFT) method, similar to that used in previously published studies [39,40,41]. The BFT method proposed by Zi et al. [40] is a modified version of the concentric ring test (ASTM C1499) for ceramic materials and a round panel test (ASTM C1550) for fiber-reinforced concrete. The small round panel sample of D420 × 48 mm^2^ used in this case shows its effectiveness in reducing the material volume, which makes it easier to conduct the test. According to the simple plate theory, the dimensions of the round panel sample were proven to ensure equibiaxial tensile behavior as reported previously [39,40]. In addition, the unchanged equibiaxial flexural stress was generated in the zone of the loading ring; thus, demonstrating its advantage for investigating the stochastic nature of the strength of the brittle material. In addition, because of the equal stress in all directions within the loading ring and the homogenous distribution of the fibers, cracks could occur in an arbitrary direction. By contrast, oriented cracks could occur if the fibers were arranged in a specific direction.

**Figure 4:**
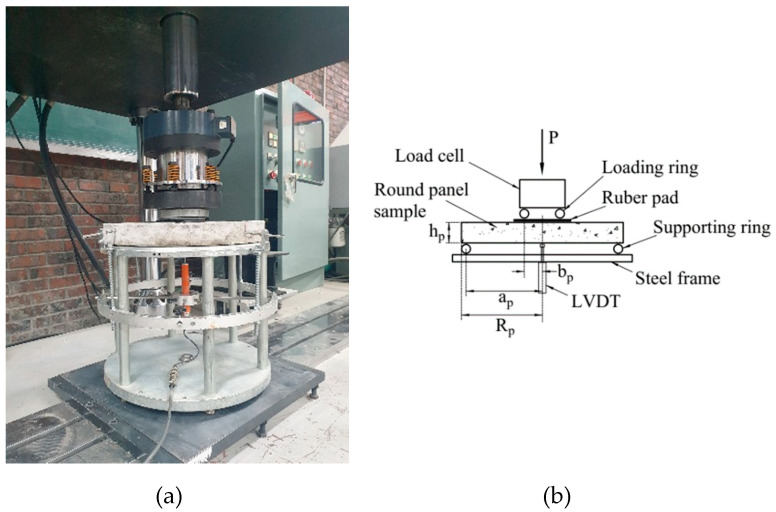
Photograph (**a**) and schematic (**b**) of the biaxial flexural test.

In this study, the BFT was conducted using a 500 kN hydraulic UTM. The round panel sample (D420 × 48 mm^2^) was supported by a steel frame. The diameter of the support ring was 400 mm (*a_P_* = 200 mm), and that of the loading ring was 100 mm (*b_P_* = 50 mm). Notably, for the high consistency of the observations, a rubber pad was placed between the sample surface and the loading ring to maintain the flat condition of the sample surface. In addition, an LVDT was attached to the steel frame to measure the deflection of the central point of the sample (see Figure 4). According to the linear elasticity theory [42], the equibiaxial flexural stress was calculated using Equation (3) as follows:

(3) $$f_{f}=\frac{3P}{4\pi h_{p}^{2}}\left\{\frac{\left(1-\nu \right)\left(a_{p}^{2}-b_{p}^{2}\right)}{R_{p}^{2}}+2\left(1+\nu \right)\ln\frac{a_{p}}{b_{p}}\right\}$$

where *P* is the applied load; *h_P_* is the panel’s thickness; ν is the Poisson’s ratio (ν = 0.184); *R_p_*, *a_p_*, and *b_p_* are the radius of the panel, support ring, and loading ring, respectively.

According to ASTM C1550 [35], the toughness at a specified central deflection was calculated using Equation (4) as:

(4) $$T=\int_{0}^{\delta }P\delta d\delta$$

where *P* and *δ* are the load and the specified central point’s deflection from the BFT, respectively.

Nine round panel samples containing three variable groups of WFN fiber-reinforced concrete (1, 2, and 3%) were prepared for the tests conducted in this study. All samples were cured in water for 28 days prior to testing. All the tests were conducted in standard laboratory conditions; the speed of loading of all tests was maintained at 1 mm/min.

## 4. Results and Discussion

### 4.1. Compressive Strength Test

**Figure 5:**
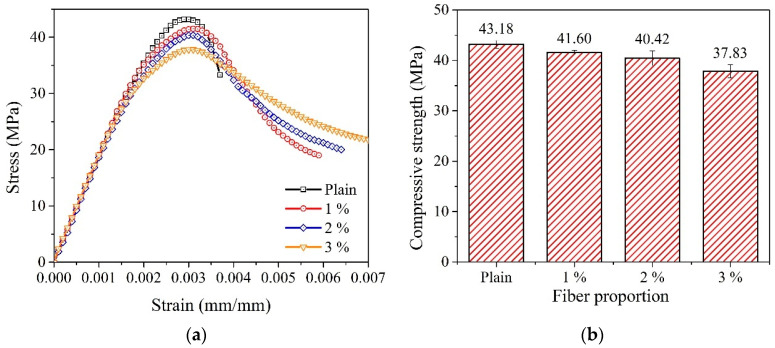
Average compressive strength results: (**a**) Constitutive curves; (**b**) compressive strength results.

**Figure 6:**
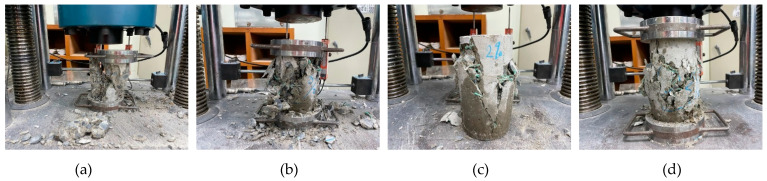
Failed cylindrical concrete samples in compressive strength test: (**a**) Plain concrete; (**b**) 1% WFN fiber-reinforced concrete; (**c**) 2% WFN fiber-reinforced concrete; (**d**) 3% WFN fiber-reinforced concrete.

The influences of the WFN fibers on the 28-day compressive behavior are shown in Figure 5. The average compressive strengths of 1, 2, and 3% WFN fiber-reinforced concrete samples and plain concrete at 28 days are listed in Table 6 (refer to Equation (1)). During the test, the plain concrete samples were broken according to a brittle scenario at the peak load, while other WFN fiber-reinforced concrete samples were broken but contained cracks around their cylindrical surfaces (see Figure 6). The WFN fibers played a role in confining the concrete. Overall, the addition of WFN fibers did not enhance the compressive strength of concrete. However, an increase in strain corresponding to an increase in the WFN fiber proportions was observed. The results in Figure 5 show slight decreases of 4, 7, and 12% in compressive strength as compared with that of plain concrete when the respective volume proportions of WFN fibers of 1, 2, and 3% were added. The comparative outcomes between the compressive strengths of concrete reinforced with 1% (by volume) polymeric and recycled polymeric fibers are listed in Table 7. The reduction in compressive behavior as compared with that of the control concrete is presented as a percentage. As shown in Table 7, the reduction in compressive strength when WFN fibers are added into the concrete is consistent with the findings from previous works [15,17,24]. In particular, the reduction in this study in the compressive strength of 4% when 1% WFN fibers were added into the concrete was most effective as compared with those reported in the literature.

According to the literature, polymeric fibers have been reported to reduce concrete compressive behavior because of the creation of additional defects in the cement matrix generated following a reduction in the matrix compactness. Consequently, the addition of polymeric fibers weakens the interfacial transition zone (ITZ) [43,44]. Fiber effectiveness was only activated after a crack occurred. Considering the post-peak stage in Figure 5a, the descending slope of the stress-strain curves is softened by the presence of fibers. In addition, an increase in peak strain was observed. These observations are clearer when the proportion of fibers is increased. Therefore, the toughness increases considerably. The toughness can be computed by the area under the stress-strain curve [45] relevant to Equation (4) and Figure 5a. The results are listed in Table 6. A study by [43] considered the effectiveness of steel fibers on the mechanical characteristics of concrete. A slight reduction in compressive strength was reported when steel fibers were added to the concrete. In addition, the role of steel fibers in softening the constitutive curve after the peak load, as well as its effect on flexural toughness, were observed. Similar findings pertaining to decreasing compressive strength and increasing strain at the peak load have been published by Choi and Yuan [46]. These authors added the same proportions (1% and 1.5%) of glass fibers and polypropylene fibers in concrete. The observation at 28 days showed a decrease in compressive strength as compared with that in the plain paste from 12 to 27%. Hence, the post-cracking behaviors of the WFN fiber concretes were similar to those reported in the literature.

According to the compressive strength test observations, the compressive strength of WFN fiber-reinforced concrete decreased with an increase in WFN fiber proportions. By contrast, toughness was enhanced. In addition, the role of the fiber in softening the constitutive curve after the peak load was clearly observed with an increase in fiber proportions.

### 4.2. Splitting Tensile Strength Test

**Figure 7:**
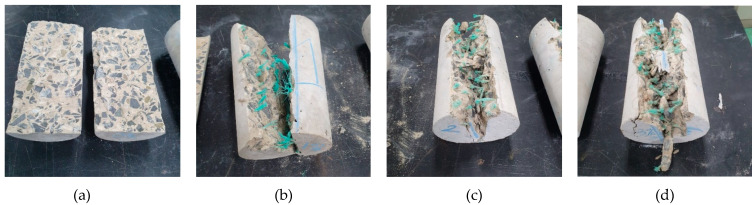
Failed cylindrical concrete samples from the splitting tensile strength test: (**a**) Plain concrete; (**b**) 1% WFN fiber-reinforced concrete; (**c**) 2% WFN fiber-reinforced concrete; (**d**) 3% WFN fiber-reinforced concrete.

**Figure 8:**
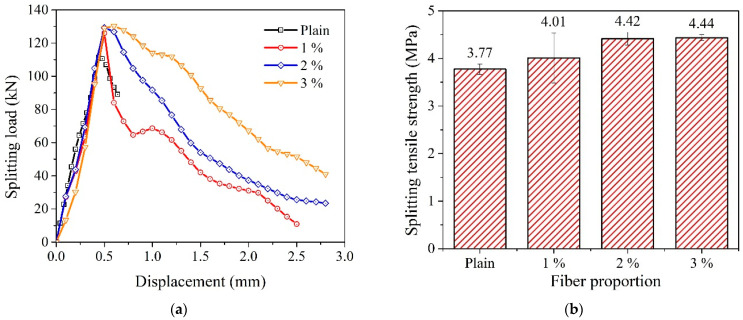
Splitting tensile strength test results: (**a**) Splitting load–displacement curves; (**b**) splitting tensile strength.

Figure 7 shows the failure samples after the splitting test. The plain concrete samples were split into two pieces. The 1% WFN fiber-reinforced concrete samples were split into two pieces, but there were several weak connections. The 2% and 3% WFN fiber-reinforced concrete samples were not split completely. Accordingly, the dense distribution of fibers inside the concrete could be observed. In addition, the original state of fiber was kept throughout the failure process, i.e., there was no cut fiber in the failed zone. The failure occurred owing to the pullout phenomena of fibers from the cement matrix. It is worth mentioning that the strain of the fiber is higher than that of the composite concrete, refer to data from Figure 2 and Figure 5a, i.e., 0.226 and from 0.003 to 0.0032 for the WFN fiber and the WFN fiber-reinforced concrete, respectively. This argument contributes to clarify that the failure phenomena were due to the pullout of the fiber from the matrix rather than the failure of the fiber itself in the matrix. The average splitting tensile strengths at 28 days for the WFN fiber-reinforced concrete and plain concrete are shown in Figure 8b and Table 6. In general, the splitting tensile strength can be computed based on the peak load (*P*) relevant to the first crack [34]. The splitting tensile strengths are presented in Figure 8b and Table 6 in compliance with Equation (2). The test results show that increases of 5, 15, and 16% can be observed as compared with that of plain concrete when the respective proportions of 1, 2, and 3% were added into the concrete. There were significant differences between the tensile strengths of the 1% WFN fiber-reinforced concrete and 2% and 3% WFN fiber-reinforced concretes; however, there was a negligible difference between the tensile strengths of the concrete containing 2% and 3% WFN fibers. Furthermore, the ductility of the concrete corresponding to the post-peak behavior of the splitting load-displacement curves increased significantly, as shown in Figure 8a. The pre-peak behaviors were similar in all materials. They increase linearly up to the first crack, which is the proportional limit that coincides with the occurrence of the first crack. The results of the splitting test of plain concrete showed no distinct behavioral trends after the peak load. By contrast, the displacement observed from other fiber-reinforced concrete samples continued to increase until failure occurred. These observations have clarified the effectiveness of WFN fibers in improving the crack resistance and the tensile strength and ductility of concrete. Once the splitting failure occurred, the fibers located in the splitting zone acted as bridging and anchoring agents that restricted the propagation of the crack, transferred the stress, and gradually withstood the applied load. The transfer of the stress of the WFN fibers improved the tensile strain capacity, as observed in the post-peak behavior in Figure 8a. Finally, an improvement in the tensile strength over the unreinforced concrete was observed. A comparative finding was published by Zhang et al. [17], which reflected the effectiveness of the use of recycled polymeric fibers for improving the splitting tensile strength of concrete. In this study, an increase of 4.3% in the splitting tensile strength of concrete was reported when 1% R-PP fibers (by volume) was added into the concrete. As shown in Table 2, the tensile strength of R-PP fibers was higher than that of WFN fibers (400 MPa as compared with 303.8 MPa, respectively); however, the response in tensile strength was reported to be lower than that in this study. Since the failure scenario depends on the pullout of the fibers from the matrix, the twisted geometry of the WFN fibers was more effective as compared with the dense and rectangular geometry of the R-PP fibers, as reported in [17].

The test results also showed that the average splitting tensile strength of WFN fiber-reinforced concrete ranged from 9 to 11% of its compressive strength. This range is similar to that found in other fiber-reinforced concrete research (glass, polypropylene, and steel fibers) [46,47].

The use of WFN fibers was effective in improving the tensile and post-cracking behavior of concrete. In addition, the failure of the fiber-reinforced concrete is observed owing to the pullout phenomena of the fiber from the cement matrix.

### 4.3. Biaxial Flexure Test

**Figure 9:**
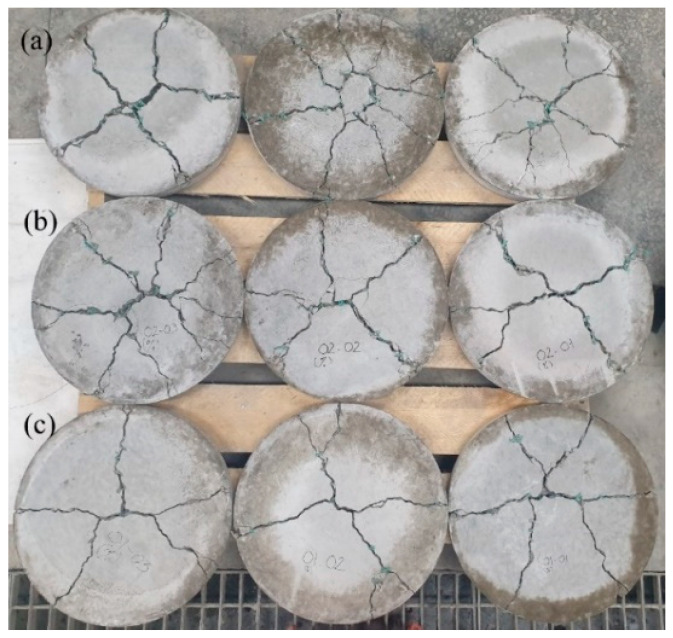
Tensile fiber of round panel concrete samples: (**a**) WFN samples with fiber-reinforced concrete proportions of (**a**) 3%; (**b**) 2%; (**c**) 1%.

**Figure 10:**
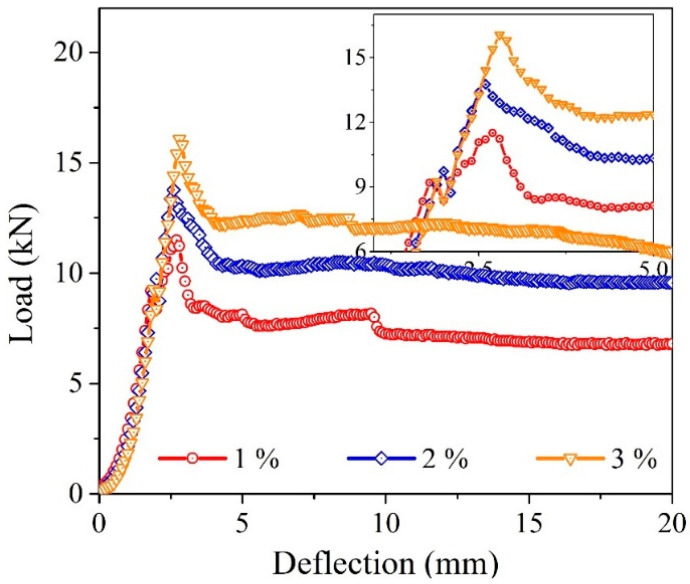
Load–deflection curves from the biaxial flexure test.

Figure 9 illustrates the failed samples from the BFT with different WFN fiber volume proportions. The deflection-hardening behavior and consistency load-deflection response were observed as shown in Figure 10 [48]. As indicated by the crack patterns, the flexural failure mode can be observed for the WFN samples with a fiber-concrete proportion of 1% (Figure 9c), while both flexural failure and punching failure modes can be observed for samples containing 2% and 3% WFN fibers (Figure 9a,b). Small and large cracks can both be observed in the failed WFN samples with fiber-reinforced concrete proportions of 2% and 3%, while only larger cracks were observed in the failed 1% WFN fiber-reinforced concrete samples. In addition, the numbers of cracks in the failed samples of the two test cases were more than that of the 1% WFN fiber-reinforced concrete sample. Thus, the role of fiber proportions is clarified. Similar to the above observations from the splitting tensile strength test, the WFN fibers showed their efficient role in bridging cracks, withstanding load, and delaying the development of crack propagations that improved the ultimate strength of materials. The higher efficiencies associated with higher fiber proportions can also be observed from these failure patterns. The literature review showed that the normal concrete samples were fractured and had no effective post-cracking ductility [39,49]. The samples were broken into two or three pieces separately, and the direction and the location of the first crack were random. When a crack occurs at peak load, the load suddenly drops to zero. Hence, this study only focused on the fiber-reinforced concrete samples.

The pre-peak stage, as illustrated in Figure 10, shows that the deformation mode depends on the elastic properties of the normal concrete. The pre-peak behaviors were similar for the three surveyed variables. Comparable effectiveness was observed with post-peak responses. Ductile behaviors can be observed owing to the addition of WFN fibers, and the improvement in this behavior is more evident with the higher WFN fiber proportions. In addition, different residual stages are observed among the three fiber proportions (3% WFN fiber-reinforced concrete >2% WFN fiber-reinforced concrete >1% WFN fiber-reinforced concrete). As the WFN fiber-reinforced concrete exhibited deflection-hardening behavior, the biaxial flexural stresses at the first crack (limit of proportionality (LOP)) and the peak (modulus of rupture (MOR)) were considered (see Table 8). At the point of LOP, the biaxial flexural stress remained unchanged when the fiber proportions were increased, and the difference between the highest and lowest stresses as compared with that of the mean stress of the three variables was approximately 6%. However, there was an increase in stress at the MOR point when increased proportions of fibers were added. As compared with the stress observed in the case of the 1% WFN fiber-reinforced concrete sample, the 2% and 3% WFN fiber-reinforced concrete samples increased by 19% and 30%, respectively.

According to ASTM C1550 [35], the toughness of fiber-reinforced concrete is determined by its energy absorption capacity, which is characterized by the areas under the load–deflection curves between the initial loading and the specified central deflection. Moreover, ASTM C 1550 [35] recommends computation of the toughness values at deflections of 5, 10, 20, and 40 mm relevant to the span length ratios of the sample, that is, at L/160, L/80, L/40, and L/20, respectively, where L = 800 mm. Thus, to comply with the standard, the central deflection points of 2.5, 5, 10, and 20, corresponding to the 420 mm diameter sample and the points of LOP and MOR, are proposed to compute the toughness in this study. The biaxial flexural strength was calculated using Equation (3), and toughness was calculated using Equation (4). The load–deflection curves are shown in Figure 10, and the biaxial flexural test results are listed in Table 8.

**Figure 11:**
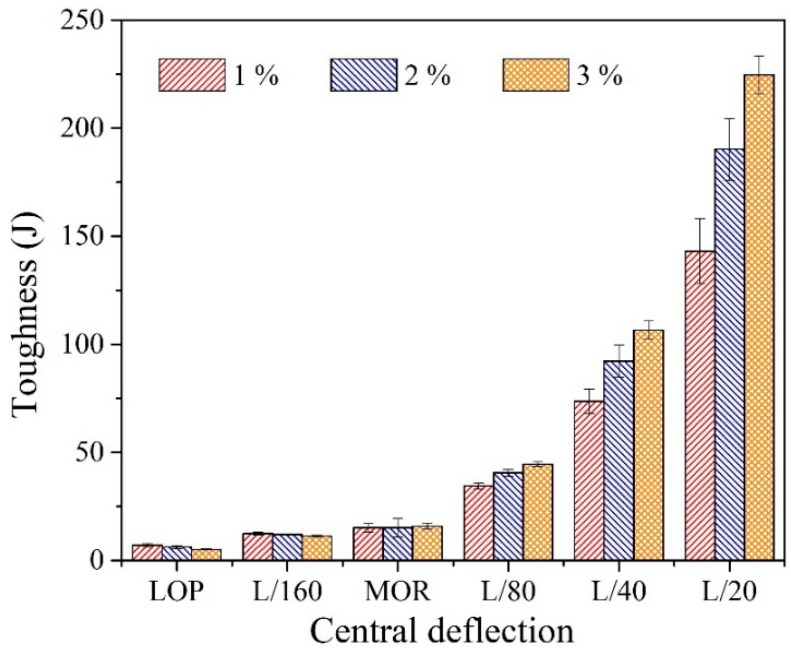
Toughness corresponding to central deflections and fiber proportions.

**Figure 12:**
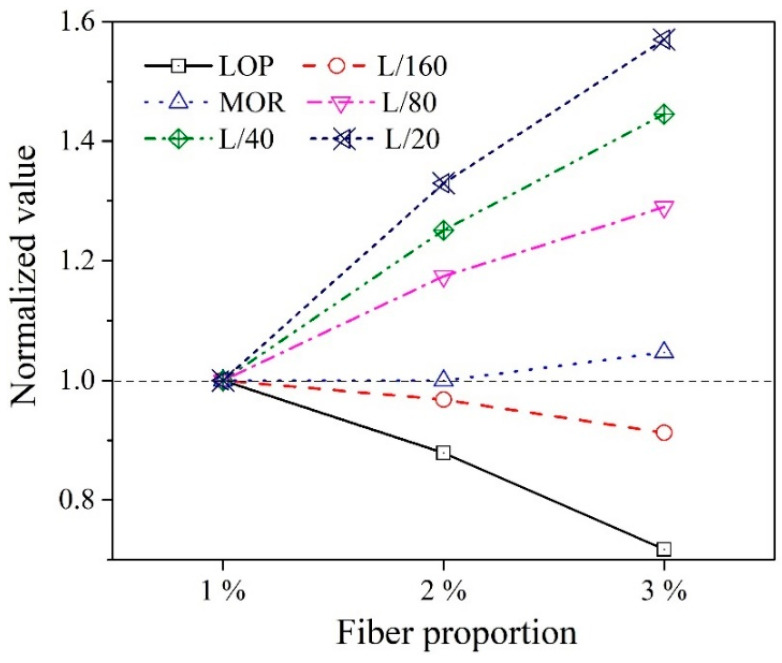
Normalized analysis based on the toughness of the 1% WFN fiber-reinforced concrete sample.

Figure 11 summarizes the toughness from the biaxial flexural test corresponding to central deflections and fiber proportions. The toughness values at the points LOP, L/160, and MOR were unchanged regardless of the fiber proportions. For instance, the mean value of toughness of each variable at the points LOP, L/160, and MOR are observed to be equal to 6, 12, and 15 J, respectively. However, different toughness values were observed at the points L/80, L/40, and L/20, and their order increased as a function of the fiber proportions. For instance, the toughness values at the points L/20 were 143, 190, 225 J, respectively. On the basis of these observations, the efficiencies corresponding to the different fiber proportions were estimated by the normalized analysis based on the toughness values of the 1% WFN fiber-reinforced concrete sample (see Figure 12). As indicated, at LOP, all the test samples showed a decrease in the toughness ratio with an increase in the proportion of fibers. This may be attributed to the fiber additions, which degraded the performance of the ITZ as reported above. A lower decrease is observed at the point L/160; thus, demonstrating the influences of fiber proportions on the toughness performance after the first crack occurred. After MOR, the toughness performances of all test samples yielded higher increases, which corresponded to the increase in fiber proportions. This is attributed to the fact that the twisted geometry of WFN fibers improved the bond between the fibers and the cement matrix. Therefore, toughness was improved at higher fiber proportions.

The toughness value at a specified central deflection was used to clarify the performance of the WFN fibers by redistributing the stress after cracking. The above findings demonstrate the effectiveness of WFN fibers in enhancing the biaxial flexural stress and toughness and the post-crack behavior of concrete.

### 4.4. Ductility Number

It is known that in a parallel system of brittle materials, if failure occurs in one part, it cannot carry the load anymore, and the entire load is transferred to the remaining parts. Conversely, if a part of a parallel system of ideally plastic materials fails, it maintains the load partially, and the other parts continue to carry the remaining load. Thus, determining the transformation of the failure mode from a brittle to quasi-brittle material or ductile is a major issue, which clarifies the improvement of material property. A previous study proposed an index as the ductility number (Equation (5)) to evaluate the failure mode of a material [50].

(5) $$\lambda =1-e^{\left[-\omega {\left(\alpha -1\right)}^{\zeta }\right]}$$

where ω and ζ are constants used to describe the relationship between λ and α (in this study, ω = ζ = 1), where is the maximum value of the peak-strain ratio.

**Figure 13:**
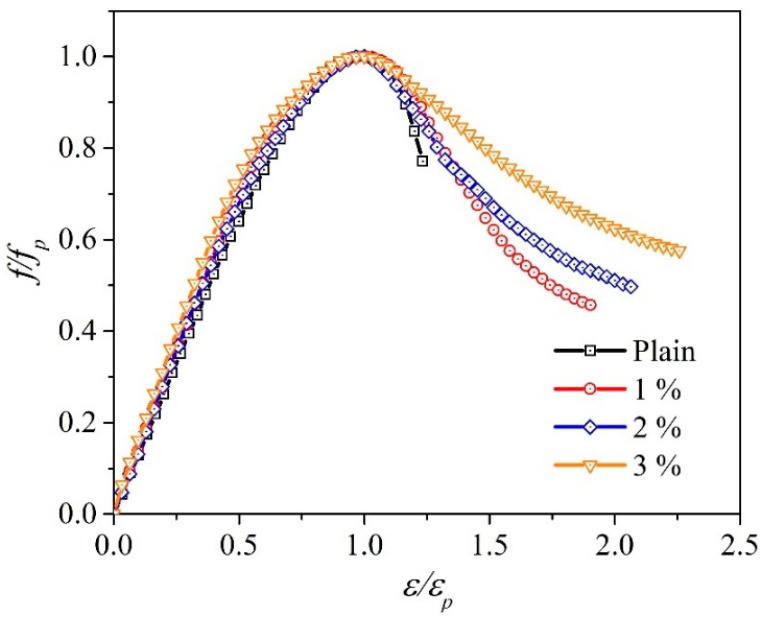
Peak-stress ratio–peak-strain ratio curves.

Figure 13 shows the relationship between the peak-stress ratio and peak-strain ratio from the compressive strength test. From the results in Figure 13, the ductility numbers are listed in Table 9.

As listed in Table 9, the ductility number increased as the fiber proportion increased. Hence, the transformation of the failure mode from a brittle to a quasi-brittle WFN fiber-reinforced concrete is clarified based on this observation.

### 4.5. Optimization of WFN Fiber Proportion

**Figure 14:**
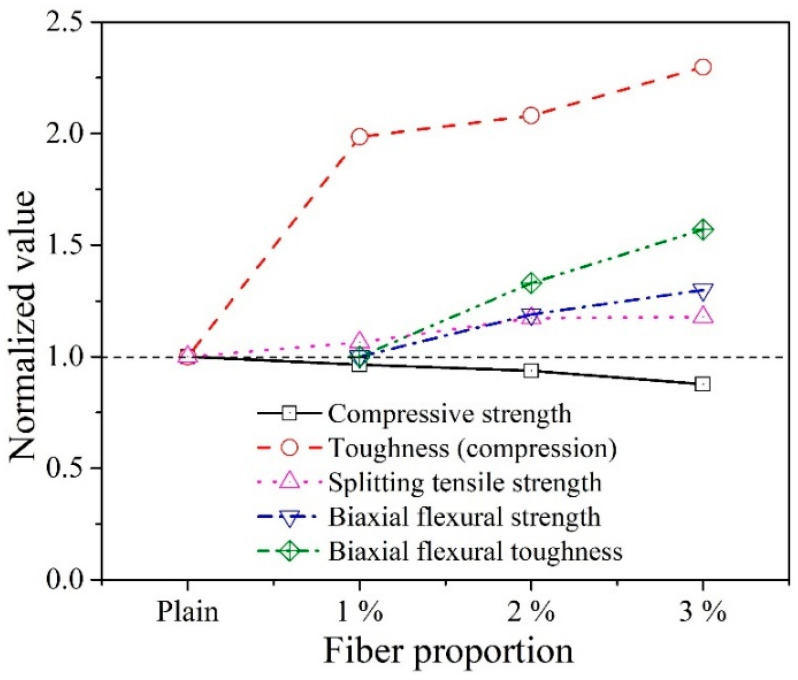
Normalized analysis of strength and toughness based on plain concrete, and biaxial flexural strength and toughness based on 1% WFN fiber-reinforced concrete sample.

The observations from this study show the effectiveness of WFN fibers in transforming the failure mode from a brittle to a quasi-brittle concrete, which is an important material property. The performance of concrete is evaluated based on compressive strength, splitting tensile strength, and biaxial flexural strength tests. As shown in Figure 14, the compressive strength decreased by 3.7% in the case of the 1% WFN fiber, while the toughness and splitting tensile strength increased by 98.5 and 6.3%, respectively. When the proportion of WFN fiber increased from 1 to 2%, the compressive strength decreased by 2.7%, while the other properties increased by 9.5 and 10.8%. In addition, the biaxial flexural strength and toughness increased by 18.9 and 33%, respectively. However, when the fiber proportion increased from 2 to 3%, the decrease in compressive strength showed a further increase, that is, 6%, while the increase in splitting tensile strength and biaxial flexural strength further decreased by 5.3 and 11%, respectively; by contrast, the toughness from compression and biaxial flexural toughness increased by 21.8 and 24%, respectively. In addition, from the material workability perspective, the slump of the 1% WFN fiber-reinforced concrete sample was acceptable as compared with those of the 2% and 3% WFN fiber-reinforced concrete samples. In addition, the findings from literature showed the cost benefit of utilizing synthetic fiber as a reinforcing agent for concrete [51,52]. Hence, using the recycled fiber from waste fishing net is a benefit not in improving material properties but also in solving the environmental and economic issues. According to these observations, the proportion of 1% WFN fibers can be considered the most economical option over the scope of the present study. In addition, the recommended proportions of 1 and 2% of WFN fibers can be applied in practice depending on the structural requirements.

## 5. Conclusions

In this study, the influences of 1, 2, and 3% proportions of WFN fibers on the strength and toughness of concrete were investigated. The following conclusions can be drawn from the observations of this study:

- The addition of WFN fibers reduced the workability of concrete, and the slump of the 1% WFN fiber-reinforced concrete sample was acceptable as compared with those of the 2 and 3% WFN fiber-reinforced concrete samples.
- Corresponding to an increase in the fiber proportions, the changes in the mechanical properties of the WFN fiber-reinforced concrete are as follows: According to the compression test, the compressive strength decreased slightly while the strain and toughness increased. According to the splitting tensile strength outcomes, an improvement in the tensile property and post-cracking behavior of concrete was observed. From the biaxial flexural test, the biaxial tensile stress and toughness improved significantly. On the basis of the findings from the experimental work, the WFN fibers showed their benefits in crack arresting, and thus improved the post-cracking behavior and transformed concrete from a brittle to a quasi-brittle material.
- On the basis of the normalized analysis, the optimal proportion of 1% WFN fibers is proposed by considering the changes in the decrease in compressive strength and the increase in splitting tensile strength and toughness among the three surveyed proportions. However, the proportions of 1% and 2% are recommended for practical applications owing to their structural requirements. In addition, further studies on detailed proportions of fiber content from 1 to 2% are necessary to find the best performance of WFN fiber-reinforced concrete material.
- The fiber utilized in this study was a recycled product from the WFN. Thus, the environmental benefits are very clear. In addition, the fiber production process does not consume any energy and releases toxic waste or exhaust. Collection, classification, washing, and cutting into desired sizes are all requirements of the production process. However, for practical applications, the demands for WFN fiber pretreatment, cutting technology, and optimal mixing design technology are necessary. Hence, these subjects will be studied in detail in the future.

In conclusion, WFN fiber is a potential reinforcement for concrete, and it can be utilized and evaluated in practical applications, especially, constructions in the marine zone, where chloride corrosion is the most concern.

## Figures and Tables

**Table 1:** Properties of waste fishing net (WFN) fibers.

Notation	Filament Diameter (mm)	Number of Filaments	Section Area (mm^2^)	Length (mm)	Tensile Strength (MPa)	Density (g/cm^3^)
WFN	0.23	42	1.74	40	303.81	0.95

**Table 2:** Comparison of tensile strength of fibers in the present study and the literature.

Property	WFN Fiber (Recycled Polyethylene (R-PE) Fiber)	Commercial PE Fiber [36]	R-Nylon Fiber [28]	R-Polypropylene (PP) Fiber [17]	R-Polyethylene Terephthalate (PET) Fiber [24]
Tensile strength (MPa)	303.8	400	348	400	420.7

**Table 3:** Mixture design for 1 m^3^ volume of concrete mix.

Notation	Water-to-Cement Ratio (W/C) (%)	Cement Type I (kg)	Water (kg)	Coarse Aggregate (4–20 mm) (kg)	Fine Aggregate (0–4 mm) (kg)	WFN Fiber (V%)	Slump (cm)
Plain 1% 2% 3%	0.45	455.3	202.9	1178.9	503.7	0 1 2 3	11 9 6 4

**Table 4:** Chemical composition and physical properties of cement.

CaO	Al_2_O_3_	SiO_2_	SO_3_	MgO	Fe_2_O_3_	Ig. Loss	Specific Surface Area (cm^2^/g)	Compressive Strength, 28-Day (MPa)
61.33	6.40	21.01	2.30	3.02	3.12	1.40	2800	36

**Table 5:** Summary of test samples.

Test	Geometry	Dimensions (mm^2^)	Number of Samples	Notation
Compressive strength	Cylinder	D100 × 200	3	Plain
			3	1%
			3	2%
			3	3%
Splitting tensile strength	Cylinder	D100 × 200	3	Plain
			3	1%
			3	2%
			3	3%
Biaxial flexural strength	Round panel	D420 × 48	3	1%
			3	2%
			3	3%

**Table 6:** Compressive strength test and splitting tensile strength test results.

	Compressive Strength (MPa)	Toughness (J/m^3^)	Splitting Tensile Strength (MPa)
Plain	43.18 (0.7541)	81,865.03 (1741.742)	3.77 (0.0668)
1%	41.60 (0.4140)	162,535.6 (5517.159)	4.01 (0.5284)
2%	40.42 (1.4626)	170,329.7 (2242.645)	4.42 (0.1491)
3%	37.83 (1.2981)	188,193.8 (2975.436)	4.44 (0.0611)

Note: Numbers in parentheses are standard deviations.

**Table 7:** Compressive behavior comparison of 1% (by volume) polymeric and recycled polymeric reinforced concrete.

	Present Work	Zhang et al. [17]	Kim et al. [24]	Ghanem et al. [15]
Fiber	WFN Fiber	R-PP Fiber	R-PET Fiber	Marco PP Fiber
Reduction (%)	4	7.1	10	21.8

**Table 8:** Biaxial flexural test results.

		Unit	1%	2%	3%
LOP	P_LOP_	kN	10.43 (0.896)	9.87 (0.208)	9.27 (0.513)
	f_fLOP_	MPa	4.3 (0.369)	4.07 (0.086)	3.82 (0.211)
	δ_LOP_	mm	1.93 (0.153)	1.97 (0.058)	1.9
	T_LOP_	J	7.11 (0.895)	6.25 (0.701)	5.1 (0.212)
L/160	P_2.5_	kN	11.07 (2.084)	13.37 (3.066)	13.27 (1.012)
	δ_2.5_	mm	2.5	2.5	2.5
	T_2.5_	J	12.48 (0.75)	12.08 (0.321)	11.39 (0.242)
MOR	P_MOR_	kN	12.6 (1.572)	14.97 (0.907)	16.37 (1.419)
	f_fMOR_	MPa	5.19 (0.648)	6.17 (0.374)	6.74 (0.585)
	δ_MOR_	mm	2.73 (0.252)	2.73 (0.321)	2.8 (0.1)
	T_MOR_	J	15.16 (1.885)	15.15 (4.289)	15.87 (1.231)
L/80	P_5_	kN	8.13 (1.15)	10.33 (1.25)	12.37 (1.002)
	δ_5_	mm	5	5	5
	T_5_	J	34.57 (1.414)	40.6 (1.611)	44.59 (1.031)
L/40	P_10_	kN	7.23 (1.429)	10.37 (1.258)	12.1 (0.361)
	δ_10_	mm	10	10	10
	T_10_	J	73.73 (5.708)	92.25 (7.595)	106.54 (4.296)
L/20	P_20_	kN	6.8 (0.854)	9.57 (0.416)	11 (1.082)
	δ_20_	mm	20	20	20
	T_20_	J	143.1 (15.074)	190.27 (14.254)	224.67 (8.699)

Note: *P*, load; *f_f_*, biaxial flexural stress; δ, deflection at the central point; T, toughness; number in parentheses, standard deviation.

**Table 9:** Ductility number results.

	Plain Concrete	1% WFN Fiber-Reinforced Concrete	2% WFN Fiber-Reinforced Concrete	3% WFN Fiber-Reinforced Concrete
α	1.23	1.90	2.06	2.26
λ	0.21	0.59	0.66	0.72

## Data Availability

The data presented in this study are available on request from the corresponding author.
